# Accessible Contraceptive Implant Removal Services: An Essential Element of Quality Service Delivery and Scale-Up

**DOI:** 10.9745/GHSP-D-16-00096

**Published:** 2016-09-28

**Authors:** Megan Christofield, Maryjane Lacoste

**Affiliations:** a Jhpiego, Baltimore, MD, USA; b Bill & Melinda Gates Foundation, Seattle, WA, USA

## Abstract

Use of contraceptive implants has surged in recent years, yet emerging data show a deficit of service delivery capacity and coverage for implant removals. The number of projected removals needed in the 69 FP2020 focus countries in 2018 (4.9–5.8 million) is more than twice that estimated for 2015 (2.2 million). We must proactively plan and execute high-quality implant removal services in order to fulfill the exceptional promise of implants in meeting client needs and advancing toward FP2020 goals.

This article was drafted on behalf of the Implants Access Program Operations Group, comprised of representatives from the Bill & Melinda Gates Foundation, the United States Agency for International Development (USAID), the United Nations Population Fund (UNFPA), and the Clinton Health Access Initiative (CHAI). It was established as a working group to proactively and reactively address operational and service delivery issues arising from increased country-level availability of implants. The overall goal of this group is to improve coordination around LARC service delivery, including investigating and addressing country operational issues arising from wider availability of implants in-country and making recommendations for the way forward to appropriate stakeholders.

Renewed investment in scaling up contraceptive implants has resulted in a dramatic increase in their use since 2012. The surge is due in part to the reduction in price and increases in donor investments made through the Implants Access Program (a collaboration between public and private organizations to make implants accessible to women in the world’s poorest countries) and ministerial prioritization and support to facilities and providers, as well as user preference. Among the 69 Family Planning 2020 (FP2020) focus countries, prevalence of injectables and implants is growing faster than all other contraceptive methods; in Ethiopia, Kenya, Malawi, Senegal, and Zimbabwe, the percentage of women ages 15–49 using implants is growing by over 1 percentage point per year.^1^ Implants are reaching more women than ever before,^2^ including those who have traditionally been underserved.^3^ Implants also now have the potential to meet the needs of postpartum women who are breastfeeding immediately after birth as a result of the World Health Organization’s recent decision to allow their use among this important population, which is reflected in the fifth edition of the *Medical Eligibility Criteria for Contraceptive Use*.^4^ Recently, implant manufacturers Merck and Bayer announced plans to sustain their current reduced implant pricing for an additional 5 years, through 2023, creating price parity for all the currently available implant products and further paving the road for potential continued scale-up.^5^

However, emerging data show that service delivery capacity for implant removals has not kept pace with that for insertion. For example, in Kenya, among Ministry of Health facilities offering family planning services in 2015, 86% provided contraceptive implants while only 67% provided removals.^6^ Furthermore, clients who access removal at private-sector (and some public-sector) facilities can encounter user fees,^7^ and those who receive their method from a mobile outreach campaign or a community health worker are at times without clear or accurate, up-to-date information on how and where to seek follow-up services and removal. While there is a paucity of evidence regarding access to removal in the peer-reviewed literature, ministries and program managers increasingly cite reports of clients’ failed attempts in obtaining the removal procedure.^8^

With the rapid expansion of implants services, the family planning community—donors, implementers, ministries, advocates, and health care providers—has reached a critical point at which it needs to assure the availability of convenient, quality removal services for clients who want removal for any reason throughout the use of their implant, including those discontinuing contraceptive use, switching to another method, or removing the implant to have a subsequent implant inserted. The family planning community has a responsibility to support method continuation as well as access to quality removal when desired—commensurate to the attention paid to the method’s initiation—such that clients’ reproductive intentions can be realized.^9^ We need more data visibility into implant removals and adoptable approaches to expanding access to removal services—and it is imperative that we act urgently.

Prevalence of implants (and injectables) is growing faster than all other contraceptive methods in the 69 FP2020 focus countries.

Service delivery capacity for implant removals has seemingly not kept pace with that for insertion.

## WHY THE FOCUSED ATTENTION ON REMOVALS?

Implant removal is an essential component of contraceptive implant scale-up, critical to offering high-quality services and continuity of care for family planning. Inadequate removal services leave some clients on contraception when they would prefer not to be, whether the intention is to conceive or discontinue the method for other reasons. This inability to access removal within a reasonable time frame consistent with access to other services compromises clients’ rights and choice. As the literature on rights-based family planning has made clear, ensuring access to implant removal helps fulfill the aim of voluntary family planning such that it extends into the method’s discontinuation as well.^10^^,^^11^ For example, the FP2020 “Rights and Empowerment Principles for Family Planning,” within the tenet of availability, states clearly, “Health care facilities, trained providers and contraceptive methods are available to ensure that individuals can exercise full choice from a full range of contraceptive methods. … Availability of services includes follow-up and removal services for implants and IUDs.”^11^

Implant removal is an essential component of implant scale-up and high-quality service delivery.

There is precedent for serious concern about lack of quality removal services. The learning generated from the global scale-up effort of Norplant implants (beginning in the early 1990s) blames the method’s low uptake, in part, on inadequate access to and quality of removals.^12^^-^^15^ Furthermore, issues of access to quality removal services had repercussions; Frost and Reich noted that “for several reasons, removal problems became major barriers to Norplant access in some countries, with negative implications for the product’s reputation, appropriate use, and customer satisfaction.”^12^ And while the advent of 1- and 2-rod implant technologies (compared with Norplant’s 6 rods) has made the removal procedure much easier,^7^ it has not freed the method from technical difficulties.^16^

The sheer volume of anticipated removals in the coming years should give us pause. This unprecedented growth in the availability of implants will result in an equal growth in the need for implant removals in the near future because currently available implants have a 3-to-5-year lifespan. Using publically available data from RHInterchange,^2^ which has contraceptive procurement data from major donors and international organizations for more than 140 countries, we modeled the approximate timeline and magnitude of this upcoming removal burden (Figure). The bars in the Figure show procurement of implants by year, over the past 10 years, in the 69 FP2020 focus countries. We calculated the lines projecting number of implants due for removal by disaggregating procurement data by implant product, adding a 12-month period from receipt in-country to insertion in a client, and then assuming that once inserted, each product was used for its couple-years of protection (CYP) unit—2.5 years for Implanon, 3.2 years for Sino-implant (II), and 3.8 years for Jadelle.^17^ For example, a shipment of Jadelle that arrived in a country in July 2008 was modeled for removal in April 2013. A second scenario is also presented in the Figure to account for the possible shift of Implanon’s qualified effectiveness from 3 years to 5 years (represented as 3.8 CYP in the model). This was applied beginning with clients who had an Implanon implant inserted in 2014, with the assumption that current users will be notified that they may keep their implant inserted longer than 3 years. Although the model uses procurement data as a proxy for use, it echoes the previously acknowledged trend of increasing growth in use of implants and conveys overall that with either scenario we will experience a growing number of removals in years to come, as current implant users age out of their implants or remove for other reasons. According to this model, the number of estimated removals needed in 2015 (2.2 million) was less than half the number projected for 2018 by either scenario (4.9 million for the first scenario, 5.8 million for the second scenario)—a worrisome figure if removal issues have already begun to emerge.

The number of estimated implant removals needed in 2015 in the 69 FP2020 focus countries was less than half the number projected for 2018.

**FIGURE.**
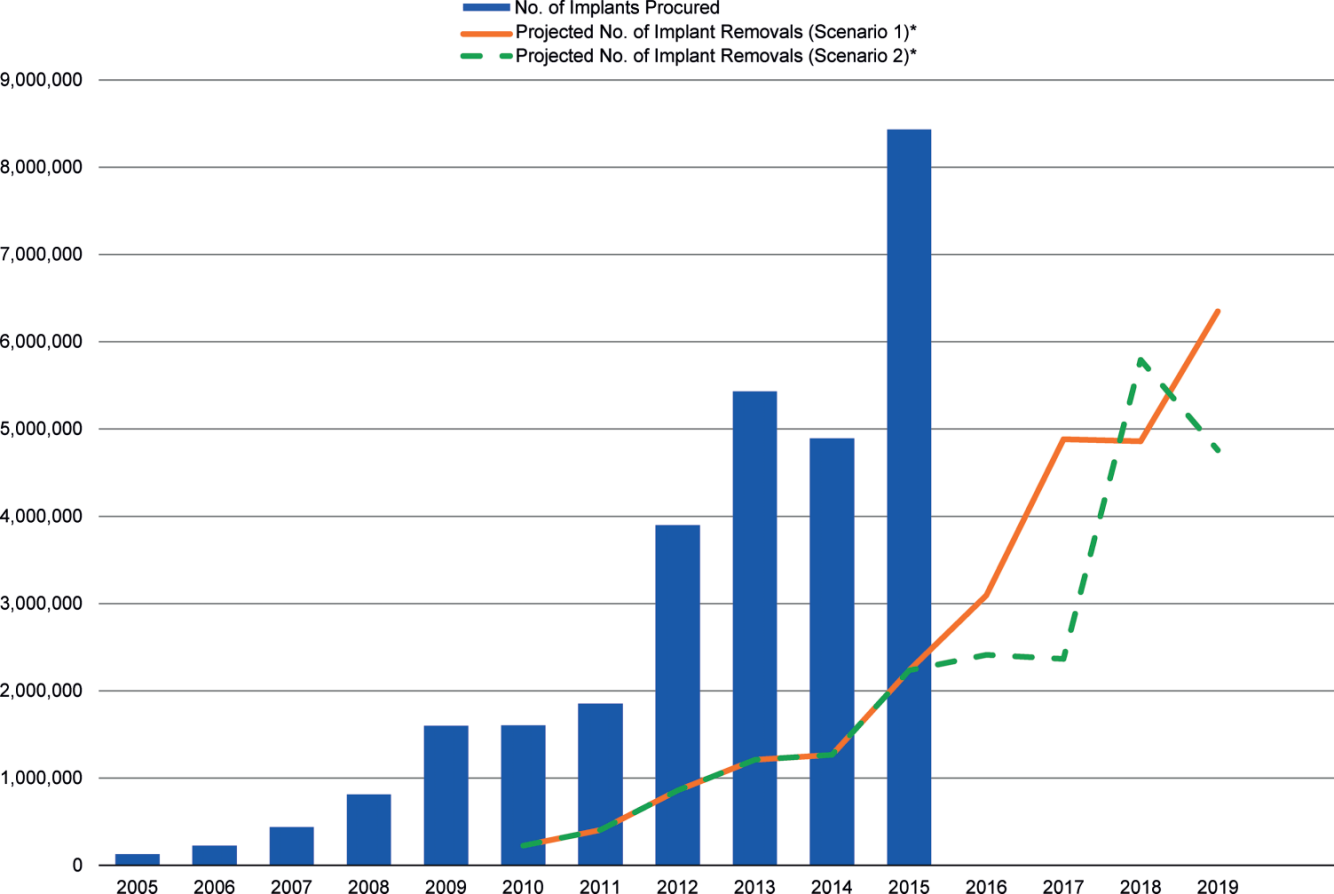
Number of Implants Procured by Year in the 69 FP2020 Focus Countries, 2005–2015, and Projected Number of Implant Removals, 2010–2019 Abbreviation: CYP, couple‐year of protection; FP2020, Family Planning 2020. * Scenario 1 assumes that each implant is used for its current CYP unit—2.5 years for Implanon, 3.2 years for Sino‐implant (II), and 3.8 years for Jadelle. Scenario 2 accounts for the possible shift in the approved length of effectiveness for Implanon, from 3 years to 5 years, which would change its CYP unit to 3.8 CYP. Source of data: RHInterchange.^2^

Of note, the model’s limitations include: (1) use of procurement data as a proxy for use, (2) the assumption that all implants are used for their CYP unit to calculate the removal projection, (3) use of the general estimate of 12 months to represent the time between the implant shipment’s arrival in country and the product’s insertion into a client, and (4) the exclusion of direct procurement by governments and some other third-party procurement (for example, Indonesia’s procurement) from RHInterchange procurement numbers.

## PROMISING EFFORTS

Access to implant removal has not been neglected entirely. In addition to the presence of removal guidance and training within national guidelines and curricula (and the global implants learning resource package^18^), many programs are addressing implant removal proactively within their independent settings. For example, with USAID funding, Marie Stopes International (MSI) – Tanzania partnered with the Ministry of Health and Social Welfare to build capacity of public-sector providers to provide voluntary long-acting reversible contraceptive services, including removal of implants and intrauterine devices (IUDs). They supported these facility-based providers by including them in a 3-week tour with an MSI mobile outreach team where they received on-the-job training with many opportunities for insertion and removal practice due to the high client load.^19^ And in Ethiopia, where health extension workers (HEWs) provide implants, helping the nation achieve great gains in the modern contraceptive prevalence rate, the Federal Ministry of Health and Pathfinder International (under the USAID-funded Integrated Family Health Program) developed a coordinated strategy to expand access to implant removal through strong referral and support mechanisms—Ethiopia’s task-shifting policy allows HEWs to insert but not remove implants.^20^^,^^21^ When HEWs identify clients in need of implant removal, they notify the linked health center, which responds by sending a skilled service provider to the community level to provide the service.^20^ In this way, clients who receive their implant from a HEW are equally supported to get the implant removed when desired. Furthermore, research is underway in Nigeria to assess the capability of community health extension workers to insert and remove implants, an assessment that has the potential to inform other community provision models. Cost solutions are also being tested, including the use of vouchers that capture the insertion, follow-up care, and removal fees within 1 voucher so that the user fee is only levied upon uptake.^22^

Some country programs are implementing innovative strategies to address implant removal.

Now is the right time to tackle the issue of ensuring ready access to high-quality implant removal services.

At a global level in 2015, several partners, including UNFPA and CHAI, developed a standardized consumables kit for contraceptive implant services. The kit includes supplies for insertion and removal, offering an easily procurable option for places where supply planning has been an issue.^5^ With Bill & Melinda Gates Foundation funding, the Performance Monitoring and Accountability 2020 (PMA2020) project and FHI 360 developed a series of implant removal access questions for piloting in PMA2020 surveys in Ethiopia and Kenya. In part, the questions aim to collect information on why and to what extent clients attempt to have their implant removed but fail to do so. Recently published data from this effort show that 4% of current implant users in Kenya and 7.2% in Ethiopia have attempted but failed to have their implant removed.^23^^,^^24^

Coordinated and systematic efforts to highlight and implement best practices in expanding access to implant removal services could greatly benefit situations in which implant removals have not received commensurate attention or are only now emerging as a problem area. At the global level, the Implants Access Program Operations Group partnered with Jhpiego to support 2 technical consultations on implant removals. In late 2015, the group initiated the Implant Removal Task Force to bring together implementing partners and donors to identify existing best practices and call attention to research and programming gaps for future action. The task force also aims to bring awareness and ensure adequate attention to the issue of implant removal. While only in its infancy, the task force has already shared lessons learned and identified a learning agenda. In addition, in each of the task force’s 4 subgroups (capacity building and service delivery; data and monitoring; research; and difficult removals), action plans have been developed and new tools, approaches, and analyses are in development to meet the needs of ministries, providers, partners, and donors. As this task force matures, it expects to deliver clear evidence and best practices and offer tangible solutions to those who need them. As a starting point, the task force has developed a short list of requirements of quality, client-centered implant removal services (Box).

BOX **What Would Quality Implant Removal Services Look Like?**

- Supplies for implant removal are available at the point of service.
- Provider is competent and confident.
- Systems are in place for managing difficult removals.
- Counseling, side‐effect management, and resupply and switching are offered.
- Client knows when and where to go for removal.
- Service is available when client wants it, within a reasonable distance.
- Service is affordable (or free).
- Removal data are collected and monitored.

Source: Compiled from the Implant Removal Task Force of the Implants Access Program Operations Group.

## WHAT WE CAN DO NOW

Now is the right time to tackle this issue. In this period of rapid scale-up of contraceptive implants, the opportunities for advocacy and action surround us. Whether in the development of costed implementation plans, in updates to heath management information systems, in the actions associated with national FP2020 commitments, or in so many other efforts, each offers the opportunity to address preparedness for, and delivery of, implant removals when desired.

First and foremost, more data are needed. The great success in implant scale-up has been measured almost exclusively by its uptake, yet monitoring removal is one way to support accountability in providing the full range of an implant service (an area where donors could add pressure by requiring reporting on revisits and discontinuation). To better assess the extent to which this full range of service—including follow-up and removal—has been scaled, programs can employ both traditional tactics, such as including follow-up and removal indicators in facility registers and survey instruments, and new ways, such as innovative monitoring of client access through technology solutions (which could potentially also capture incidence of difficult removals and indicators of quality). Although an increasing number of countries track implant uptake through DHIS 2 and other health management information systems, few routinely track removals; yet systematic capture of data on removals is integral to developing plans and scaling up access to this essential service. So too, we need to better understand reasons for method switching and discontinuation.^25^ Altogether, these data could offer ministries and program managers visibility into the performance of their family planning programs, which will help them align volumes of insertions with volumes of removals, and could reflect the quality of care within their programs. With this is mind, the data and monitoring subgroup of the Implant Removals Task Force is developing an adaptable tool to assist countries in identifying and addressing implant removal trends and issues. Additionally, where possible globally managed surveys, such as those conducted by PMA2020 and the Demographic and Health Surveys, should incorporate questions on capacity to provide removal services, number of implant removals, their timing of removal in relation to the 3-to-5-year life-of-use, the reasons for removal, and whether the client elected to use another implant or any other method after removal of an implant.

Ultimately all inserted implants will need to be removed, and thus the ability to offer quality removal services on demand is of inevitable importance. This readiness rests on our ability to:

1. **Ensure clients are well-informed.** Not only should women be counseled at the time of insertion on when, where, and how to access follow-up care and removal,^26^ but programs should also explore how to make certain this information is available on an ongoing basis and clients are aware when it is time to have the implant removed. Community mobilization, including use of community health workers, as well as social and behavior change communication efforts may be effective here.
2. **Support providers to maintain competence and confidence in the removal procedure.** We must find ways to overcome the barriers to providers’ ability to perform implant removals, through traditional and non-traditional approaches. Currently, opportunities for clinical practice are limited because demand for removals at training events is relatively low or nonexistent during this phase of introduction or rapid scale-up. This means few providers get the chance to practice in the supportive learning environment of a training. Those who *do* leave training competent may face low client load upon return to their facility, which leaves them with few opportunities to maintain their skill.^7^ These factors may also affect the *quality* of the removal and capacity to identify and manage difficult removals. Reviewing training plans (including plans for client mobilization) and expanding session times on removal practice is a starting point while additional solutions are explored.
3. **Plan ways to include implant provision (insertion, follow-up, and removal) within the total health system.** At a planning level, strategies, budgets, and costed implementation plans should accommodate the equipment and consumables, human resources, and trainings required to ensure availability of removal services, including ensuring that services are free for the poor or that any fees levied are affordable. While it may not be feasible for every level of the health system or provider offering implants to provide removals, planning will be needed to establish removal services through a referral system or other approaches. Additionally, service delivery approaches that expand access to implants should include contingencies for access to removal services. For example, task shifting and mobile outreach have helped implants meet demand for family planning and penetrate farther into rural and other underserved communities, but these programs must think ahead when expanding access through means that do not guarantee continuous and universal access to a competent provider for follow-up and removal. An optimal approach to expanding access to implant removals likely involves a total market approach that leverages private and NGO partners and uses their comparative advantages to ensure that the needs of all segments of the population are met and that available resources are maximized so the poor and vulnerable are not left out.

The current scale-up of implants is the result of coordinated efforts on the part of many groups and individuals, including providers, seeking to sustain the expanded choice of methods presented by implants. To prevent the ongoing scale-up from being undermined, further investment and efforts to measure and expand access to implant removal are needed, and the timing is right. We also call on advocates to aid in focusing efforts and refining our “asks” at the global, national, and subnational levels, such that change can be achieved thoughtfully and efficiently.

The Implant Removal Task Force is poised to play an important role in collecting and disseminating knowledge on this topic; we encourage all parties engaged in contraceptive implants provision to look carefully at all aspects of providing quality removal services—the client, the provider, and the system—and take swift action to ensure the availability of quality implant removal services.

## Notes

**Peer Reviewed**

**First Published Online:** 2016 Aug 30
